# Growth Differentiation Factor 15 and Physical Function Impairment in the SardiNIA Study

**DOI:** 10.3390/jcm15072612

**Published:** 2026-03-29

**Authors:** Nicia I. Profili, Edoardo Fiorillo, Valeria Orrù, Maria Benelli, Francesco Cucca, Alessandro P. Delitala

**Affiliations:** 1 Department of Medicine, Surgery, and Pharmacy, University of Sassari, 07100 Sassari, Italy; nicia.isa.profili@gmail.com (N.I.P.); mariabenelli9@gmail.com (M.B.); 2 Institute for Genetic and Biomedical Research, National Research Council, 09042 Monserrato, Italy; edoardo.fiorillo@cnr.it (E.F.); valeria.orru@cnr.it (V.O.); fcucca@uniss.it (F.C.); 3 Department of Biomedical Science, University of Sassari, 07100 Sassari, Italy

**Keywords:** GDF-15, grip strength, gait speed, skeletal muscle, reduced physical performance, mitochondrial dysfunction

## Abstract

**Background:** Sarcopenia is the age-related, progressive loss of strength, function, and skeletal muscle mass, which can be assessed with specific tests. The Growth differentiation factor 15 (GDF-15) has been proposed as a key biomarker of aging, and it has been associated with mitochondrial dysfunction, cachexia, and physical impairment. **Methods:** The cohort of this study comes from the SardiNIA study, an ongoing longitudinal survey focused on the identification of genetic and phenotypic variants associated with aging. We assessed hand grip strength, gait speed, and GDF-15 in all samples. Linear multivariate analysis was used to assess the correlation after adjusting for a range of potential confounders. **Results:** The sample consisted of 4842 subjects (57.5% female) with a median age of 48.6 years. Levels of GDF-15 were comparable between males and females and showed a strong positive association with aging (rho 0.617, *p* < 0.001). Linear multivariate regression analyses showed that GDF-15 was negatively associated with gait speed and grip strength in both hands (respectively, Beta −0.09, Beta −0.07, and Beta −0.08, *p* < 0.001 for all). **Conclusions:** GDF-15 was negatively associated with physical function. GDF-15 may be considered a proxy for reduced physical performance. Future research is needed to understand the pathogenetic role of GDF-15 in the reduction in skeletal muscle in aging people.

## 1. Introduction

Sarcopenia is the age-related decline in muscle mass, physical function, and strength. It has a high frequency in older subjects and is linked to increased frequency of chronic diseases, functional decline, and mortality [1]. The role of skeletal muscle in the body homeostasis is not limited to physical activity but influences health with different mechanisms such as regulating glucose and insulin metabolism [2]. Loss of skeletal muscle starts from the fourth decade of life [3], dramatically increases during aging and can be as high as 40% in elderly people [4]. It can be further influenced by different factors, such as reduced physical activity, inappropriate nutrition, and chronic diseases [5]. Sarcopenia, which is associated with frailty, is commonly used to describe the significant loss of skeletal muscle. However, in 2010 the European Working Group on Sarcopenia in Older People pointed out that term sarcopenia should be used in case of decreased muscle strength and reduced physical performance assessed with gait speed and handgrip strength [6]. Different tests can be used to assess sarcopenia, whose selection depends on patient mobility, resource of health care, and the purpose of testing. The exact pathogenetic mechanism of sarcopenia is still not understood, but evidence suggests that the key mechanisms are chronic inflammation, hormonal dysregulation, and mitochondrial dysfunction [7]. The current view is that all these mechanisms act synergistically. Indeed, the increase in reactive oxygen species can exert direct damage to skeletal muscle by promoting oxidation and degradation of the protein, and may lead to mitochondrial dysfunction [8], thus contributing to the loss of muscle [7]. Sarcopenia has a critical impact in clinical setting and is associated with increased risk of negative outcomes such as disability, hospitalization, and mortality [9].

Growth differentiation factor-15 (GDF-15) is a member of the transforming growth factor-β (TGFβ) superfamily and primarily acts in the area postrema and nucleus tractus solitarius where via the GDNF Family Receptor Alpha-Like REarranged (GFRAL) during Transfection receptor modulates appetite and energy balance [10]. In normal condition, it has a low expression in different tissues, but it can be increased by specific stimuli. Indeed, GDF-15 is released in response to pro-inflammatory cytokines, which induced its mRNA expression and could act as an autocrine inhibitor of inflammation. GDF-15 is further upregulated by hypoxia and anoxia in cancer cells [11]. In addition, it has been proposed as a key biomarker of aging, and it has been associated with mitochondrial dysfunction, cachexia, and physical impairment [12,13,14]. GDF-15 increased with aging and is associated with mitochondrial dysfunction in skeletal muscle and loss of muscle in aged mice [15]. GDF-15 also correlates with systemic inflammation and functional decline [16], and it has been associated with declining physical function in a cohort of elderly patients [17]. Its potential role in reducing physical performance has been confirmed in a longitudinal study over 9 years of follow-up [18]. A recent meta-analysis reported that GDF-15 levels were higher in sarcopenic subjects, and analysis also demonstrated that the mild association between GDF-15 and sarcopenia was somewhat limited by the limited number of studies [19]. Most of the previous studies included a small sample of patients, often limited to older subjects, without accounting for major confounders. Therefore, in this study we aimed to test the correlation between GDF-15 and continuous physical performance measures (gait speed and handgrip strength) as a proxy of sarcopenia in a cohort of subjects with a wide age span from SardiNIA study.

## 2. Materials and Methods

We used data from SardiNIA study, a longitudinal cohort study started in 2001 and focused on the identification of genetic and phenotypic variants associated with aging. The initial cohort consisted of 6148 subjects aged 14–102 years, which represented roughly 62% of the eligible population of four neighboring towns (Lanusei, Arzana, Ilbono, and Elini) in Sardinia, Italy. Participants completed the first survey between 2001 and 2004 and were invited to join the project every 3–4 years, thus generating 5 complete surveys.

In each survey, the medical visit was performed by a trained physician and a nurse and included a full 2 h evaluation, including a medical history interview, collection of anthropometric measurements, and assessment of specific cardiovascular parameters as well as geriatric tests. Details of the projects can be found elsewhere [20]. To this study, we used data from the third set of visits.

### 2.1. Assessment of Variables

Blood pressure was measured three times in the morning after 5 min of resting period. The mean of the last two measurements was used in the analysis. Weight, height, and waist circumference were collected in all subjects. Body Mass Index (BMI) was calculated by dividing body weight in kilograms by height in square meters. We defined hypertension as the presence of either of systolic blood pressure (SBP) ≥ 140 mmHg and/or diastolic blood pressure (DBP) ≥ 90 mmHg and/or self-reported use of anti-hypertensive drugs. Diabetes was defined in all patients who self-reported a diagnosis of diabetes mellitus, and/or in case of fasting glycemia ≥ 126 mg/dL and/or glycated hemoglobin (HbA1c) ≥ 6.5%. Current consumers of at least one cigarette per day were considered smokers. Study methods were conducted according to the principles expressed in the Declaration of Helsinki. The protocol was approved by the governing Ethics Committee, Azienda Sanitaria Locale 4, and each participant signed a written informed consent.

### 2.2. Physical Performance Measures

The participants were asked to wear comfortable clothing and shoes and were positioned behind a marked starting. The examiner first demonstrated the gait speed test. Subjects were instructed to walk at their “usual speed” and start walking when prompted. They were told to keep walking past the finish line. Walking speed was calculated as gait speed length divided by time to complete the course (m/s). The time was recorded using a chronometer. Grip strength was measured by a handheld Baseline dynamometer (Fabrication Enterprises Inc., Elmsford, NY, USA). During the test, the participant was comfortably seated on a chair with elbow leaning on the table. Three trials were performed for each hand, with a 20 s resting interval between trials. Values were separately tabulated and the average values for each hand were used for analyses.

### 2.3. Laboratory Assays

Blood venous samples were drawn in the morning after overnight fasting. Serum GDF-15 levels were measured by ELISA (DY957 R&D Systems Inc., 614 McKinley Place N.E. 55413 Minneapolis, MN, USA), as previously reported [21]. GDF-15 was assessed in the whole sample except for 124 subjects due to lack of serum. Total cholesterol and cholesterol were quantified using an enzymatic method (Abbott Laboratories ABA-200 ATC Biochromatic Analyzer, Irving, TX, USA). High-density lipoprotein (HDL) cholesterol was measured with a dextran sulphate–magnesium precipitation. Low-density lipoprotein (LDL) cholesterol was calculated with the following Friedewald formula: LDL cholesterol = total cholesterol − [HDL cholesterol + (triglycerides/5)]. Fasting plasma glucose concentration was quantified by using the glucose oxidase method (Beckman Instruments Inc., Fullerton, CA, USA).

### 2.4. Statistical Analysis

We tested the normality of continuous variables with the Shapiro–Wilk test. Due to the skewed distribution, data were reported as median and interquartile range. Accordingly, the Wilcoxon rank sum test was used to assess the difference between continuous variables among groups, while the Spearman correlation test was used to compare two continuous variables. Pearson χ^2^ was used to compare the differences among frequencies. To test the effect of GDF-15 on grip strength and gait speed, we created three different multivariable linear regression models, in which covariates found significantly associated at univariate analysis were treated as independent variables, without account for relatedness, while GDF-15 was included as continuous variable. We first included variables associated in the univariate analysis and those not associated with the outcome were removed. Sex was treated as covariate in the model because its interaction with GDF-15 interaction was not significant. The final “best model” contained only the covariates which led to a statistically significant increase in R^2^ (purposeful selection). Collinearity was reported when values of variance inflation factor (VIF) were greater than 10. *p*-value < 0.05 indicated statistical significance in STATA version 18.0.

## 3. Results

Summary characteristics of the sample are summarized in Table 1. Females were younger, had a lower waist circumference and BMI than males (respectively, *p* = 0.04, *p* < 0.001 and *p* < 0.001) who had higher levels of glycemia and HbA1c (*p* < 0.001 for all), and had a higher frequency of diabetes. Males were more likely to have dyslipidemia (33.85% vs. 19.93%, *p* < 0.001) and have higher triglycerides than females (*p* < 0.001). Levels of LDL were comparable between males and females, who had higher total cholesterol and HDL concentrations (respectively *p* = 0.01 and *p* < 0.001). Males had also higher frequency of hypertension (39.28% vs. 28.09%, *p* < 0.001) and higher levels of both SBP and DBP (*p* < 0.001 for all). Females had a lower grip strength and lower gait speed than male (*p* < 0.001 for all). The concentration of GDF-15 was similar between the two groups.

**Figure 1:**
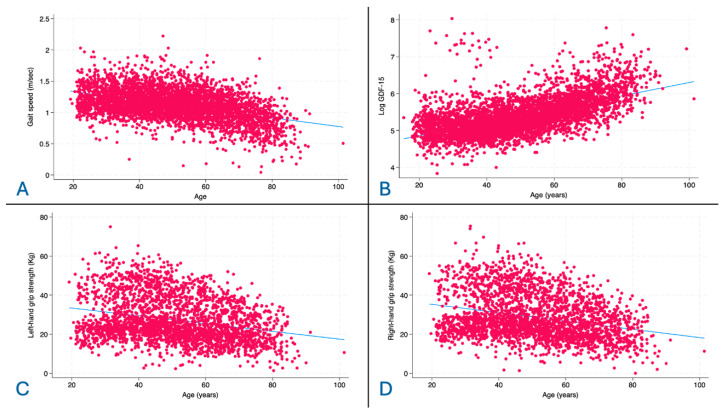
(**A**): correlation between gait speed and Age. (**B**): correlation between log GDF-15 and age. (**C**): correlation between left-hand grip strength and age; (**D**): correlation between right-hand grip strength and age.

Figure 1 shows the variation in gait speed (panel A), GDF-15 (panel B), left-hand grip strength (panel C), and right-hand grip strength (panel D) in the cohort as a continuous function of age. Gait speed and grip strength (right and left hand) were all negatively associated with aging (respectively, rho −0.437, rho −0.271, rho −0.258, *p* < 0.001 for all). GDF-15 showed a strong positive association with aging (rho 0.617, *p* < 0.001).

**Figure 2:**
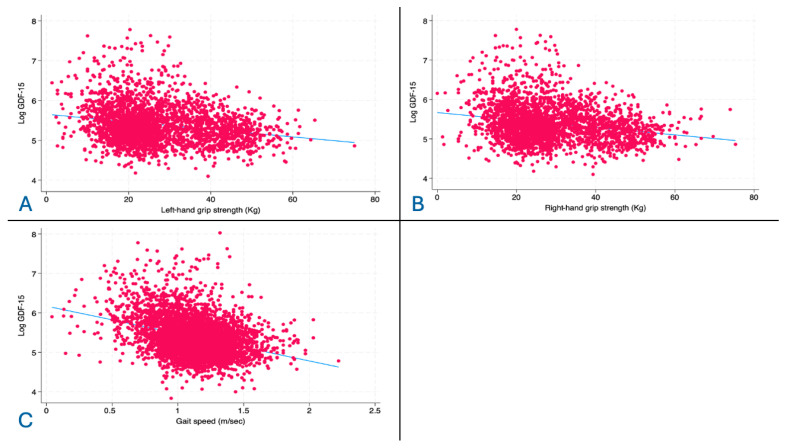
(**A**): correlation between log-transformed GDF-15 and left-hand grip strength. (**B**): correlation between log-transformed GDF-15 and right-hand grip strength. (**C**): correlation between log-transformed GDF-15 and gait speed.

Figure 2 illustrates the correlation between GDF-15 and gait speed (panel A), left-hand grip strength (panel B), and right-hand grip strength (panel C). GDF-15 was negatively correlated with all tests (respectively, rho −0.311, rho −0.204, rho −0.209, *p* < 0.001 for all). 

Table 2 reports the results of multivariate regression analysis of gait speed. Age, BMI, SBP, and GDF-15 were negatively correlated with gait speed (*p* < 0.001 for all), while DBP showed a positive correlation (Beta 0.130, *p* < 0.001).

Table 3 shows the results of multivariate regression analysis of right-hand grip strength. After adjusting for confounders age, sex, BMI, smoke, DBP, diabetes, and hypertension, GDF15 showed a negative association with right-hand grip strength (Beta −0.07, *p* < 0.001)

Table 4 reports the determinants of left-hand grip strength. GDF15 was negatively associated with the exposure variable (Beta −0.08, *p* < 0.001), after adjusting for age, sex, BMI, DBP, and hypertension.

## 4. Discussion

In this study, we found that GDF-15 was negatively associated with gait speed and hand grip strength.

Several methods have been validated to assess physical performance in aging people, and, among these, gait speed and grip strength are commonly used in clinical practice [22]. They are both connected to the concept of sarcopenia, which encloses the decline in muscle strength, muscle, and function found in the elderly [23]. The health of the skeletal muscle is critical to increase the health span. Indeed, muscle represents over 40% of body weight and contributes to specific functions other than contraction of movement, such as thermogenesis and glucose metabolism [6]. The decrease in muscle strength and mass is not linear. Indeed, it starts to decline during the fourth decade and becomes steeper in subsequent years [3]. It has been estimated that the annual loss in muscle mass in subjects aged at least 75 is extremely high: 0.6–0.7% in females and 0.8–1.0% in males [24]. The loss of skeletal muscle is heterogenous, and the severity of sarcopenia may vary in the different muscles and is further affected by exercise and diet [3,25]. Hand grip strength and gait speed are tests commonly performed in the evaluation of elderly people, but despite their clinical importance, they often suffer from limited precision [26]. Hence, biomarkers help to promote early diagnosis and to predict clinical evolution [27]. GDF-15 has recently emerged as a key role in aging, although its exact role in this context is not clear.

Previous studies showed the interaction between GDF-15 and reduced muscular function [28], which, in turn, may lead to reduced physical performance [14]. Gait speed and grip strength can be considered as a proxy of physical performance, and a proteomic study showed that are associated with GDF-15 [14]. Grip strength and gait speed are components of physical frailty, as proposed by Fried [29], and a GWAS study from UK biobank clearly showed that frailty was associated with GDF-15, which represented one of the top five markers most closely related [30]. Epigenetic alterations confirmed the importance of GDF-15. Indeed, Zou et al. found that subjects in the highest tertile of DNA methylation-estimated GDF-15 levels had an increased frailty risk (odds ratio 1.46) [31]. Further, cubic splines analysis showed a liner dose–response association with frailty. Consistently, other studies reported association between GDF-15 and muscle strength in patients with cardiovascular disease [32], idiopathic pulmonary fibrosis [33], and patients receiving cardiovascular surgery [33,34]. Further, GDF-15 showed a strong association with gait speed and hand grip strength and with all-cause mortality in a cohort of community-dwelling older adults [35].

However, there are also studies which failed to report an association between GDF-15 and sarcopenia. Indeed, a study of older Asian adults reported a null association between GDF15 and muscle-function related parameters [36]. Similarly, the study by Sanchez-Sanchez et al. showed that GDF-15 was not associated with an increased frequency of sarcopenia nor with its evolution over 2 years of follow up [37]. Another study tested whether physical activity in older adults might change levels of GDF-15 [38]. Authors showed that after 16 weeks of physical activity intervention, neither gait speed nor GDF-15 changed significantly from baseline to the end of protocol study. On the contrary, mouse models showed that cachectic mice treated with anti-GDF-15 antibody had body weight gain, improved muscle function, and physical performance [39].

GDF-15 acts at different levels and mediates different pathways, but it is still not known how it interacts with muscle weakness [40]. Thus, we can speculate different mechanisms. GDF-15 expression is not limited to skeletal muscle, but is also expressed in gastrointestinal organs, endometrium, endothelial cells, myocytes, and vascular smooth muscle cells. The finding of the increased GDF-15 levels in patients with mitochondrial myopathies [41] strengthened the hypothesis that GDF-15 is linked to mitochondrial dysfunction, which could be the link between loss of skeletal muscle and GDF-15. Mitochondria are the source of cellular energy and when dysfunctional they produce excess or altered reactive oxygen species, which causes cellular damage and contributes to systemic inflammatory response [42]. The decline in mitochondrial activity is one of the key elements found in skeletal muscle and mouse models of mitochondrial myopathy treated with a GDF-15 neutralizing antibody can ameliorate the loss of skeletal muscle and physical performance [43]. Another possibility could be that the increased levels of GDF-15 found in aging subjects have no pathogenetic role but, instead, they are due to the reduced mitochondria activity. Animal studies are consistent with this hypothesis. Indeed, Chen et al. showed that the expression of GDF-15 was selectively increased in skeletal muscle but not in other organs in aged mice, which had impaired insulin sensitivity and mitochondrial activity [15]. Finally, we can speculate that the role of GDF-15 in reduced physical performance is not limited to the loss of skeletal muscle, but it can also have additional effects. Indeed, GDF-15 was found to be negatively associated with hemoglobin and with hepcidin [44], thus contributing to the impaired physical performance.

We acknowledge that the cross-sectional design of this study does not allow for the establishment of causal relationships or the analysis of changes over time. In addition, we did not test as covariates glomerular filtration rate, which is strongly associated with serum GDF-15 levels. Finally, our cohort is middle-aged and only 20% of the sample was aged at least 65 years.

## 5. Conclusions

In conclusion, our analysis based on a cross-sectional analysis of a large sample of community-dwelling outpatients showed that GDF-15 was negatively correlated with hand grip strength and gait speed. These tests are components of sarcopenia and, therefore, our finding could suggest the role of GDF-15 as a proxy of reduced muscle mass. However, the cross-sectional design does not allow us to draw cause–effect relationships. Future research is needed to understand the pathogenetic role of GDF-15 in the reduction in skeletal muscle in aging people.

## Figures and Tables

**Table 1:** Descriptive characteristics of the sample.

	Female	Male	Total	*p* Value
n	2795	2047	4842	
Age, yrs	48.3 (36.6–61.3)	49.4 (37.4–63.5)	48.6 (37–62.2)	0.04
Age ≥ 65 years, n (%)	539 (19.3%)	458 (22.4%)	997 (20.1%)	0.005
Waist, cm	82 (75–90)	93 (86–100)	87 (78–95)	<0.001
BMI, Kg/m^2^	24.3 (21.4–27.9)	26.6 (24.2–29.3)	25.5 (22.5–28.7)	<0.001
HbA1c, %	5.3 (5–5.6)	5.4 (5–5.7)	5.3 (5–5.6)	<0.001
Glycemia, mg/dL	90.4 (84.7–98.3)	96.65 (89.6–107.0)	92.89 (86.3–102.0)	<0.001
Total cholesterol, mg/dL	215 (190–242)	212 (183–240)	214 (187–241)	0.01
LDL, mg/dL	133 (112–155)	135 (110–160)	133 (111–157)	0.31
HDL, mg/dL	60 (52–69)	49 (43–57)	55 (47–65)	<0.001
Triglycerides, mg/dL	89 (66–124)	111 (81–157)	98 (72–138)	<0.001
GDF-15, pg/mL	205.2 (158.2–279.3)	205.0 (155.7–290.5)	205.1 (156.8–282.2)	0.55
SBP, mmHg	117 (107–130)	126 (117–137)	120 (110–133.5)	<0.001
DBP, mmHg	73 (67–80)	78 (71–85)	75 (69–81.5)	<0.001
Grip strength right, Kg	22 (18.33–25.67)	39.67 (32.67–46.33)	26 (20.33–36.33)	<0.001
Grip strength left, Kg	20.67 (17–24)	38 (31.33–44.67)	24.33 (19.33–34.67)	<0.001
Gait speed, m/s	1.09 (0.95–1.24)	1.15 (1.03–1.31)	1.12 (0.98–1.27)	<0.001
Diabetes, n (%)	104 (3.72%)	103 (5.03%)	207 (4.28%)	0.03
Dyslipidemia, n (%)	557 (19.93%)	693 (33.85%)	1250 (25.82%)	<0.001
Hypertension, n (%)	785 (28.09%)	804 (39.28%)	1589 (32.82%)	<0.001

Continuous variables are expressed as median and interquartile range. Abbreviation: BMI, body mass index; HbA1c, glycated hemoglobin; LDL, low density lipoprotein cholesterol; HDL, high density lipoprotein cholesterol; GDF-15, Growth differentiation factor-15; SBP, systolic blood pressure; DBP, diastolic blood pressure.

**Table 2:** Multivariate regression analysis of gait speed.

	Beta	Std. Err.	t	*p* Value
Age	−0.39	0.001	−22.65	<0.001
Sex	0.18	0.007	12.68	<0.001
BMI	−0.09	0.001	−5.34	<0.001
SBP	−0.10	0.001	−4.35	<0.001
DBP	0.13	0.001	6.30	<0.001
GDF-15	−0.09	0.001	−6.27	<0.001

Abbreviation: Beta, beta coefficients; Std. err., standard error; t, *t*-test; BMI, body mass index; SBP, systolic blood pressure; DBP, diastolic blood pressure; GDF-15, Growth differentiation factor-15.

**Table 3:** Multivariate regression analysis of right-hand grip strength.

	Beta	Std. Err.	t	*p* Value
Age	−0.32	0.01	−22.62	<0.001
Sex	0.75	0.27	65.24	<0.001
BMI	0.06	0.03	4.22	<0.001
Smoke	0.02	0.37	2.00	0.046
DBP	0.08	0.02	5.51	<0.001
Diabetes	−0.03	0.69	−2.49	0.013
Hypertension	−0.05	0.36	−3.56	<0.001
GDF-15	−0.07	0.01	−5.72	<0.001

Abbreviation: Beta, beta coefficients; Std. err., standard error; t, *t*-test; BMI, body mass index; DBP, diastolic blood pressure; GDF-15, Growth differentiation factor-15.

**Table 4:** Multivariate regression analysis of left-hand grip strength.

	Beta	Std. Err.	t	*p* Value
Age	−0.31	0.01	−22.22	<0.001
Sex	0.76	0.26	67.1	<0.001
BMI	0.05	0.03	4.25	<0.001
DBP	0.07	0.02	5.33	<0.001
Hypertension	−0.06	0.35	−4.02	<0.001
GDF-15	−0.08	0.01	−6.65	<0.001

Abbreviation: Beta, beta coefficients; Std. err., standard error; t, *t*-test; BMI, body mass index; DBP, diastolic blood pressure; GDF-15, Growth differentiation factor-15.

## Data Availability

The data generated in this study are available upon request from the corresponding author.
